# Efficacy of the Oral Fluorouracil Pro-drug Capecitabine in Cancer Treatment: a Review 

**DOI:** 10.3390/molecules13081897

**Published:** 2008-08-27

**Authors:** Georgios V. Koukourakis, Vassilios Kouloulias, Michael J. Koukourakis, Georgios A. Zacharias, Haralabos Zabatis, John Kouvaris

**Affiliations:** 1 Attikon University Hospital of Athens, 2^nd^ Radiology Department, Radiation Therapy Unit, Medical School of Athens, Greece; Emails: gkoyokoyrakis@yahoo.gr (Koukourakis); vkouloul@cc.ece.ntua.gr (Kouloulias); 2 University Hospital of Thrace, Radiation Therapy Unit, Alexandroupolis, Greece; Email: targ@her.forthnet.gr; 3 Policlinic of Athens, Section of Pathology, Athens Greece. Email: george_zaharias@yahoo.gr; 4 Saint Savvas Anticancer Institute of Athens, 1^st^ Radiation Therapy Unit Athens Greece; Email: bzabatis@hol.gr; 5 Aretaieion University Hospital, 1^st^ Radiology Department, Radiation Therapy Unit, Medical School of Athens, Greece; Email: johnkouv@aretaieio.uoa.gr

**Keywords:** Capecitabine, Xeloda, cancer treatment

## Abstract

Capecitabine (Xeloda®) was developed as a pro-drug of fluorouracil (FU), with the aim of improving tolerability and intratumor drug concentrations through its tumor-specific conversion to the active drug. The purpose of this paper is to review the available information on capecitabine, focusing on its clinical effectiveness against various carcinomas. Identification of all eligible English trails was made by searching the PubMed and Cochrane databases from 1980 to 2007. Search terms included capecitabine, Xeloda and cancer treatment. Nowadays, FDA has approved the use of capecitabine as a first line therapy in patients with metastatic colorectal cancer when single-agent fluoropyrimidine is preferred. The drug is also approved for use as a single agent in metastatic breast cancer patients who are resistant to both anthracycline and paclitaxel-based regimens or when further anthracycline treatment is contraindicated. It is also approved in combination with docetaxel after failure of prior anthracycline-based chemotherapy. In patients with prostate, pancreatic, renal cell and ovarian carcinomas, capecitabine as a single-agent or in combination with other drugs has also shown benefits. Improved tolerability and comparable efficacy, compared with the intravenous FU/LV combination, in addition to its oral administration, make capecitabine an attractive option for the treatment of several types of carcinomas.

## Introduction

5-Fluorouracil (5-FU) is the fluorinated analog of uracil which was first synthesized in 1957. It belongs to the antimetabolite family and is a chemotherapeutic agent with activity against a variety of solid tumours, including head and neck, breast, prostate, pancreatic, liver, genitourinary and gastrointestinal tract carcinomas. When combined with radiation therapy, improved local control and survival rates have been reported in a variety of malignancies, conpared to radiotherapy alone [1].

This drug has a complex molecular activity, interfering with DNA synthesis and mRNA translation. Thymidine phosphorylase transforms 5-FU to 5-fluorodeoxyuridine (5FdUrd), which binds to thymidilate synthase and to tetrahydrofolate, forming a stable complex and thus preventing the formation of thymidine from thymine. DNA synthesis is blocked, leading to cell death. 

Moreover, through the enzymatic activity of thymidine kinase, the 5FdUrd can be metabolized into fluorouridinemono- and triphosphate (FdUMP and FdUTP), which is directly inserted into the DNA, leading to pathological DNA structures. The mRNA polymerase can also use FdUTP for mRNA formation, leading to blockage of the mRNA translation. 

Due to the fact that 5-FU has an unpredictable gastrointestinal absorption and rapid degradation, it must be administered intravenously. 5-FU concentrations in plasma depend on drug dosage as well as the rate of administration, because it displays saturable pharmacokinetics [2]. In colorectal cancer patients, it was found that protracted infusion of 5 to 28 days increased the response rate (RR) from 14% (achieved with bolus infusions) to 22% [3]. 

Nevertheless, continuous-infusion of 5-FU has been complicated by hospital and/or home health costs, infection risk of IV devices, and overall patient burden [4]. To overcome these disadvantages, still preserving the benefits of continuous-infusion, oral pro-drugs of FU were developed.

The first oral 5-FU prodrug, namely Ftorafur (Tegafur), was developed in 1967 and a Phase I study in patients with gastrointestinal carcinomas showed palliative benefits. Nevertheless, further development of that product in the United States was limited due to neurologic toxicities [1]. UFT, a combination of tegafur and uracil, an inhibitor of the primary enzyme responsible for FU degradation to central nervous system active metabolites, is currently being evaluated [1]. 

**Figure 1:**
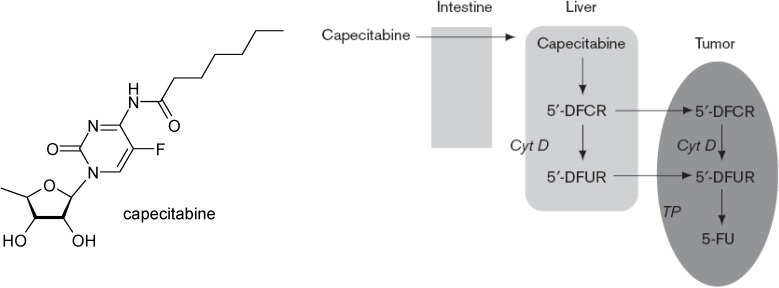
Three-step metabolic conversion of capecitabine to fluorouracil (FU).

Another oral pro-drug that takes advantage of a different metabolic pathway to form 5-FU, is doxifluridine (5'-FdUrd; 5'-deoxy-5-fluorouridine). The enzyme thymidine phosphorylase is necessary to convert this pro-drug to its active form. Higher levels of this enzyme are expressed in tumour tissues and the intestinal tract, which is responsible for dose limiting toxicity evidenced by diarrhea [5,6]. Capecitabine is a carbonate derivative of 5'-DFUR that is absorbed through the intestine in pro-drug form. Three activation steps are necessary to metabolize capecitabine to its active form FU (Figure 1). Capecitabine is absorbed through the intestine and converted to 5'-deoxy-S-fluorocytidine (5'-DFCR) by carboxylesterase and then to 5'-deoxy-S-fluorouridine (5'-DFUR) by cytidine deaminase (Cyt D), both steps taking place in the liver. Finally, thymidine phosphorylase(TP) converts S'-DFUR to the active drug, FU. This occurs in both tumor and normal tissues; however, the enzyme is found at higher concentrations in most tumor tissue compared with normal healthy tissue. This theoretically allows a selective activation of the drug and low systemic toxicity [7,8]. 

The purpose of this study is to review the available information on capecitabine with respect to clinical effectiveness on different types of cancer, adverse profile effects, dosage and administration. Data from studies using capecitabine, in combination with other chemotherapeutic agents are also reported.

## Materials and Methods

### Identification of Eligible Studies

We searched MEDLINE and the Cochrane Central Register of Controlled Trials (last search on December 2007) using combinations of terms, such as: capecitabine, Xeloda and cancer treatment. We considered all meta-analyses or randomized controlled trials providing information about the effectiveness of capecitabine on cancer treatment of different anatomical sites, adverse profile effects, dosage and administration, and future directions of ongoing research as eligible.

### Data Extraction

We extracted information from each eligible study. The data recorded, included author’s name, year of publication, number of patients included in the study, combination(s) of drugs used, doses of drugs, percentage overall response, median time to progression and median survival.

### Dosage and administration

Capecitabine is available in 150 mg or 500 mg tablets. The FDA-approved dose for a first line treatment of metastatic colorectal cancer in patients whose single agent fluoropirimidine therapy is preferred is 1,250 mg/m^2^ and given orally twice per day, usually separated into 12 hours for the first 2 weeks of every 3-week cycle. In patients with metastatic breast cancer who have developed resistance to both anthracycline and taxane based regimens or in whom any further anthracycline therapy is contraindicated, the FDA approved dose for single agent therapy is the same as for colorectal cancer patients. Additionally, the approved combination regimen with docetaxel for metastatic breast cancer patients, in whom prior anthracycline therapy has failed, is 1,250 mg/m^2^ capecitabine administered orally twice a day for the first 2 weeks with 75 mg/m^2^ docetaxel as a 1-hour IV infusion on the first day of every 3-week cycle. The product data recommends the tablets to be administered within 30 minutes of a meal; although this may decrease gastrointestinal discomfort, it may also decrease a systemic exposure to the drug [9]. 

Based on tolerability, dosage modifications are recommended. When grade 1 adverse events take place, a dosage reduction is not necessary. However, modifications are recommended on higher grade toxicities. Capecitabine should be stopped until resolution of the event to grade 1 or less, for all toxicities from grade 2. Depending on the severity and frequency of the adverse event, the drug may be restarted from 100% to 50% of the original dose. Once dosage reduction has taken place, it must not be increased again. Capecitabine may be discontinued permanently after the fourth appearance of grade 2 toxicity, third appearance of grade 3 toxicity or after any evidence of grade 4 toxicity [9].

### Safety and tolerability

Table 1 shows the incidence of adverse effects experienced on 758 patients with breast or colorectal cancer included in 3 trials. All patients received 2,500 mg/m^2^ capecitabine per day in 2 divided doses for 14 days, followed by 1 week without drug. The average duration of therapy was 127 days. The side effects that occurred in >25% of patients included anaemia, diarrhea, hand-foot syndrome, nausea, hyperbilirubinemia, fatigue/weakness, abdominal pain, vomiting, and dermatitis. A total of 8% of the 162 breast-cancer patients discontinued treatment, due to adverse events or intercurrent illness [9].

## Results

The US Food and Drug Administration (FDA) has approved capecitabine for use as a first line therapy in patients with metastatic colorectal cancer, when single-agent fluoropirimidine therapy is preferred. The drug is also approved for use in metastatic breast-cancer patients as a single agent following resistance to either anthracycline- and paclitaxel-based treatments or when anthracycline administration is contraindicated. Moreover, capecitabine is also approved in combination with docetaxel after failure of prior anthracycline based chemotherapy [9]. Its role in the treatment of patients with renal cell, pancreatic, ovarian and prostate cancer has been also evaluated. 

**Table 1:** Incidence of adverse effects in 758 patients with breast and colorectal cancer based on the evidence of three trails. All patients received 2,500 mg/m^2^ capecitabine per day in 2 divided doses for 14 days, followed by 1 week without drug.

Side effect	%	Side effect	%	Side effect	%
Taste disturbance	6	Oral discomfort	12	lymphopenia	20
Chest pain	6	GI motility disorder	12	Stomatitis-	27
Alopecia	6	Dizziness	12	Anorexia	28
Ileus	6	ArthraIgia/Myalgia	12	Dermatitis	30
Gastro-intestinal hemorrhage	6	Headache	13	Vomiting	30
Thrombocytopenia	7	Dyspnea	14	Abdominal pain-	32
Cough	8	Neuropathy/Paresthesia	15	Fatigue/Weakness	42
Venous thrombosis	9	Eye irritation	15.5	Hyperbilirubinemia	42.5
Dehydration	10	Edema	15.5	Nausea	44
Skin/Nail discoloration	10	Constipation	16	Hand foot syndrome	55
Insomnia	11	Neutropenia	17	Diarrhea	55.5
Back pain	12	Pyrexia	18	Anemia	78

The primary end point in many studies was objective RR (response rate), which was demonstrated with radio-logic examination after completion of the treatment. That was defined as the total number of patients achieving complete response (CR) or partial response (PR). CR was defined as disappearance of all known diseases at all involved sites. PR was defined as the presence of residual disease with a >50% decrease in the diameter of lesions. Progressive disease was defined as an increase of >25% in the diameter of lesions or the appearance of new lesions. Stable disease was reported when there was no change in the lesions or a probable change was not categorized as CR, PR, or progressive disease [10]. 

### Colorectal cancer

For more than four decades, the principal treatment for advanced or metastatic colorectal cancer was based on FU, used as a single agent in combination with leucovorin (LV), or in association with newer drugs, such as irinotecan or oxaliplatin [11]. In patients with metastatic disease, capecitabine has been studied as a single agent in two Phase III trials in direct comparison with FU/LV for first line therapy and in no comparative studies with irinotecan and oxaliplatin [10,12,13,14,15,16,17,18,19,20,21,22]. 

Two randomized, non-blinded Phase III trials were conducted comparing capecitabine and IV FU/LV as a first-line therapy in patients with metastatic colorectal cancer [10,12]. The trials were identical with respect to study design, primary and secondary end points, patients’ inclusion and exclusion criteria, conduct, and monitoring. The first study was conducted at 61 centres throughout the United States, Canada, Brazil and Mexico and enrolled 605 patients [10]. The second was conducted at 59 centres throughout Europe, Australia, New Zealand, Taiwan and Israel [12]. The objective tumour RR was the primary end point and was demonstrated that capecitabine was at least as active as FU/LV in inducing tumour responses. This evaluation was done by primary investigators as well as an Independent Review Committee (IRC) consisting of a panel of blinded radiologists who evaluated tumour response solely based on imaging. Time to progression (TTP) and overall survival (OS), duration to response, time to first response, time to treatment failure, safety and quality of life were secondary endpoints. Patients were randomized through a computerized randomization system to receive either capecitabine or 5-FU/LV. Capecitabine (1,250 mg/m^2^) was taken orally within 30 minutes of food twice a day for 2 weeks of treatment followed by 1 week of rest. 

The Mayo Clinic regimen was used in the FU/LV arm (LV 20 mg/m^2^ as a rapid iv. injection followed by 5-FU 425 mg/m^2^ as a bolus injection every day from day 1 to day 5; cycles repeated every 4 weeks). If the patient didn’t show disease progression or unacceptable toxicity, the treatment would be continued until the scheduled 30-week assessment. In patients showing response to treatment or in those with stable disease, treatment might be extended beyond 30 weeks at the prudence of the investigator [10,12]. Regarding the extent and site of metastatic disease as well as baseline prognostic indicators, the two arms were well balanced in both studies with the exception of a higher alkaline phosphatase concentration in the capecitabine group in the study by Hoff *et al.* [10]. The overall RRs were 26% versus 17% (P < 0.001) when evaluated by the investigators, and 22% versus 13% (P < 0.001) when assessed by the IRC, with both rates favouring the capecitabine arms. Subgroup analysis showed a higher RR for capecitabine-treated patients who had received adjuvant therapy before the trial (21.1% vs 9.0%, P < 0.05), for patients with predominantly lung metastasis (33.3% vs 10.3%, P < 0.05), and for those with only 1 metastatic site (37.8% vs 21.8%, P < 0.05). The median duration of treatment was similar between the 2 therapies: 4.5 months for capecitabine and 4.6 months for 5-FU/LV. Median time to response was shorter in the capecitabine patients (1.7 months vs 2.4 months, P value not reported). These benefits did not translate into an improvement of TTP or OS, however. The median TTP was 4.6 months in the capecitabine group and 4.7 months for 5-FU/LV (P = 0.95), with no baseline characteristics demonstrating any significant differences. Median survival rates were 12.9 months and 12.8 months for the capecitabine and FU/LV groups, respectively. As far as the toxicity profile is concerned, the followed results were observed in favour of the capecitabine arm: diarrhea 47.7% vs. 58.2%, stomatitis 24.3% vs. 61.6%, alopecia 6.0% vs. 20.6%, grade 3-4 neutropenia 2.3% vs. 22.8% and neutropenic fever 0.2% vs. 3.4%. Hand-foot syndrome occurred more frequently in the capecitabine groups (53.5% vs. 6.2%). Dose reductions due to toxicity of capecitabine were necessary in 27.3% of patients in the study by Van Cutsem *et al.* [12] and in 40.5% of patients in the study by Hoff *et al.* [10]. 35.1% and 49.3% of the patients receiving 5-FU required dose reductions in the respective studies. Dose reduction was necessary due to the hand-foot syndrome and diarrhea in the group of capecitabine, while diarrhea and stomatitis were the main causes of dose reduction in the 5-FU/LV arm [10,12,13]. 

The role of LV, when combined with 5-FU, is to stabilize the tertiary complex with thymidylate synthase and prolong the cytotoxic effect of active drug [1]. The effect of LV with capecitabine was evaluated in a single Phase II study [14]. Patients with advanced colorectal cancer were randomized to receive intermittent therapy (2 weeks on treatment, 1 week off) with either capecitabine alone (1,255 mg/m^2^ twice daily, n = 34) or capecitabine (828 mg/m^2^ and LV 30 mg/d, both dosed twice a day, n = 35). Overall RRs were 24% in the single-agent arm and 23% in the LV arm (P values not reported). Median TTP favoured the single-agent group (230 days vs 165 days). The capecitabine/LV combination produced more diarrhea (any grade: 44% vs 57%; grade 3 or 4: 9% vs 20%) and hand-foot syndrome (any grade: 44% vs 55%; grade 3: 15% vs 23%). Combined dosing with LV did not provide added benefit in terms of RR or TTP and produced more adverse events [14]. 

In patients with metastatic colorectal cancer, combinations of 5-FU/LV with the camptothecin irinotecan or the platinum analog oxaliplatin have produced encouraging RRs and are often used as first line therapy [11]. In numerous non-comparative Phase II studies, both of these drugs have been evaluated in combination with capecitabine (Table 2) [15,16,17,18,19,20,21,22]. 

**Table 2:** Phase II studies combinations of Capecitabine with irinotecan or oxaliplatin in metastatic colorectal cancer.

Author, year publication	Number of patients	Combination used	Doses of drugs	% overall response (CR+PR)	mTTP Months	Median Survival Months
Cassidy *et al.* [15], 2004	96	Capecitabine	-2000 mg/m ^2^ per day (days 1-14)	55	7.7	19.5
		Oxaliplatin	-130 mg/m ^2^ day 1			
Zeuli *et al.* [16], 2003	43	Capecitabine	-2500 mg/m ^2^ per day (days 1-14)	44	-	20
		Oxaliplatin	-120 mg/m ^2^ day 1			
Borner *et al.* [17], 2002	43	Capecitabine	-2500 mg/m ^2^ per day (days 1-14)	49	5.9	17.1
		Oxaliplatin	-130 mg/m ^2^ day 1			
Shields *et al.* [18], 2004	35	Capecitabine	-1500 mg/m ^2^ per day (days 1-14)	37,1	-	NR
		Oxaliplatin	-30 mg/m ^2^ day 1			
Bajetta *et al.* [19], 2004	68	Capecitabine	-2500 mg/m ^2^ per day (days 2-15)	47	8.3	-
		Irinotecan	-300 mg/m ^2^ day 1			
Bajetta *et al.* [19], 2004	66	Capecitabine	-2500 mg/m ^2^ per day (days 2-15)	44	7.6	-
		Irinotecan	-150 mg/m ^2^ days 1and 8			
Patt *et al.* [20], 2004	52	Capecitabine	-2000 mg/m ^2^ per day (days 2-15)	46	7.1	15.6
		Irinotecan	-250 mg/m ^2^ day 1			
Cartwright *et al.* [21], 2004	49	Capecitabine	-2000 mg/m ^2^ per day (days 2-15)	45	5.7	13.4
		Irinotecan	-240 mg/m ^2^ day 1			
Kim *et al.* [22], 2003	43	Capecitabine	-2000 mg/m ^2^ per day (days 2-15)	46,6	NR	NR
		Irinotecan	-100 mg/m ^2^ days 1and 8			

*Abbreviations*: CR - complete response; PR - partial response; mTTP - median time to progression; NR - not reached. All capecitabine doses were divided equally and dosed twice daily. Regimens were administered every 3 weeks.

Oxaliplatin has been shown to up regulate thymidine phosphorylase, which may result in synergistic activity with capecitabine [15]. Although there was not a direct comparison between the two therapies, the combination of capecitabine and oxaliplatin yielded similar results to that of FU/LV and oxaliplatin in terms of overall RR ( 37-55% vs 34-49%, respectively) and median survival (17-20 months vs 16-21 months, respectively) [11,15,16,17,18]. 

Moreover, the most common adverse effects were oxaliplatin-related sensory neuropathy, nausea and vomiting, and capecitabine-related diarrhea [15,16,17,18]. Yet, although the two therapies were not directly compared, the irinotecan/capecitabine combination also yielded similar results to FU/LV plus irinotecan with respect to overall RR (44%-47% vs 39%-54%, respectively) and median survival (13.4-15.6 months vs 14.8-20 months, respectively) [11,19,20,21,22] The most common adverse effects were diarrhea, nausea, vomiting, and neutropenia [19,20,21,22]. Phase III comparative trials are needed to establish the future role of these combinations in the first line treatment of colorectal cancerIn patients with locally advanced rectal cancer, the addition of chemotherapy to preoperative radiotherapy increases the amount of downstaging and thus improves local control. The evidence that the addition of chemotherapy to preoperative radiotherapy improves local control rates has recently been shown by two separate trials. The EORTC 22921 trial which randomized between preoperative radiotherapy (45 Gy) versus preoperative chemoradiotherapy (45 Gy combined with 5-FU/Leucovorin). The results demonstrated an increased local control rate for the chemoradiation arm: 91% versus 83% [23,24]. A similar result was found in the French FFCD 9203 study, which randomized between preoperative radiotherapy (45 Gy) and preoperative chemoradiotherapy (45 Gy and 5-FU/Leucovorin) and which showed local recurrence rate of 16.5% and 8%, respectively [25]. The fact that the orally administered capecitabine may be more effective and less toxic than IV 5-FU, has been investigated in several Phase II trials [26,27,28,29,30,31,32,33,34], (Table 3). All trails have shown that preoperative chemoradiotherapy using capecitabine achieved encouraging downstaging and sphincter preservation with a low toxicity profile. 

Kim *et al.* [35] has compared the effects of treatment with oral capecitabine vs. bolus 5-FU, administered concurrently with preoperative radiotherapy in patients with locally advanced rectal cancer (LARC). One hundred and twenty-seven patients with LARC received concurrent preoperative chemoradiation using two cycles bolus 5-FU (500 mg/m^2^/day) plus leucovorin (LV, 20 mg/m^2^/day) (Group I).

**Table 3:** Phase II trials of preoperative chemoradiotherapy in patients with locally advanced colorectal cancer.

Author, year publication	Number of patients	Treatment	Doses of drugs and radiation therapy	Complete response	Downstaging	Tolerability
Dupuis [2007], [26]	51	-Radiotherapy -Capecitabine	-45 Gy/25 fractions -825 mg/m^2^/bid throughout RT	20%	48%	no grade 4 toxicity
Desai [2007] , [27]	30	-Radiotherapy -Capecitabine	-50.4 Gy/1.8 Gy day -1330 mg/m^2^/day in 2 divided doses throughout RT	11%	37%	no grade 4 toxicity
Korkolis [2007] , [28]	30	-Radiotherapy -Capecitabine	-50.4 Gy/1.8Gy day -825 mg/m^2^/bid/ throughout RT	23%	84%	no grade 4 toxicity
Willeke [2007] , [29]	36	-Radiotherapy -Capecitabine -Irinotecan	-50.4 Gy/1.8 Gy day -500mg/m^2^ bid ( days 1-38) -50mg/m^2^ weekly	15%	41%	Grade 4 leucopenia in 2 patients
Velenik [2006], [30]	57	-Radiotherapy -Capecitabine	-45 Gy/25 fractions/1.8 Gy -1650 mg/m^2^/day in 2 divided doses throughout RT	9.1%	49.1%	no grade 4 toxicity
Krishnan [2006], [31]	54	-Radiotherapy -Capecitabine	-52.5 Gy/30 fractions -825 mg/m^2^/bid/throughout RT	18%	52%	no grade 4 toxicity
De Paoli [2006], [32]	53	-Radiotherapy -Capecitabine	-50.4 Gy/1.8 Gy day -825 mg/m^2^/bid/ throughout RT	24%	57%	no grade 4 toxicity
Machiels [2005] , [33]	40	-Radiotherapy -Capecitabine -Oxaliplatin	-45 Gy/25 fractions/1.8 Gy -825 mg/m^2^/bid/throughout RT -40 mg/m^2^ weekly for 5 weeks	14%	32%	grade 3/4 toxicity 30%
Kim [2005] [34]	95	-Radiotherapy -Capecitabine	-50 Gy/25 fractions -1650 mg/m^2^/day in 2 divided doses throughout RT	12%	71%	no grade 4 toxicity

*Abbreviations*: RT: radiation therapy, bid: twice daily

Another LARC group with 97 patients received concurrent chemoradiation using two cycles 1,650 mg/m^2^/day of oral capecitabine and 20 mg/m^2^/day of LV (Group II). Radiation was delivered to the primary tumor at 50.4 Gy in both groups. Definitive surgery was performed 6 weeks after the completion of chemoradiation. A pathologic complete remission was achieved in 11.4% of patients in Group I and in 22.2% of patients in Group II (p= 0.042). The down-staging rates of the primary tumor and lymph nodes were 39.0/ 68.7% in Group I and 61.1/87.5% in Group II (p=0.002/0.005). Sphincter-preserving surgery was possible in 42.1% of patients in Group I and 66.7% of those in Group II (p=0.021). Grade 3 or 4 leucopenia, diarrhea, and radiation dermatitis were statistically more prevalent in Group I than in Group II, while the opposite was true for grade 3 hand-foot syndrome. Preoperative chemoradiation using oral capecitabine was better tolerated than bolus 5-FU and was more effective in the promotion of both down-staging and sphincter preservation in patients with LARC. Additional larger Phase III trials are needed to better clarify these promising results of combination preoperative chemo-radiotherapy using capecitabine in patients with LARC.

### Breast cancer

In patients with breast cancer capecitabine has been investigated for use as a first line therapy and in pre-treated patients with advanced and metastatic disease, as a single agent and in combination with other chemotherapeutic agents, most often a taxane [36,37,38,39,40,41,42,43,44,45,46]. The main part of these trials was of Phase II and provides preliminary data on TTP and objective RR. Table 4 summarises the larger Phase II trial results of Capecitabine in patients with advanced and metastatic breast cancer [37,38,39,40,41,42,43,44,45,46]. 

In patients with advanced breast cancer, the tolerability and overall RR of single agent Capecitabine was compared with CMF as a first line therapy in a randomized, open label, Phase II trial [39]. The prior administration of an antiestrogen was not an exclusion criterion. Patients were randomized in a 2:1 ratio to receive either capecitabine 2510 mg/m ^2^ per day in 2 divided doses on days 1 through 14 of a 21-day cycle (n = 61) or CMF (cyclophosphamide 600 mg/m ^2^ IV, methotrexate 40 mg/m ^2^ IV, and fluorouracil 600 mg/m ^2^ IV) once every 3 weeks (n = 32). The overall RR in the capecitabine group was 30%, with 3 CRs, whereas the CMF patients had a 16% overall RR, with no CRs. Time to response was faster in patients receiving capecitabine, with 13 of the 18 responders having evidence of response by week 6 compared to none of the 5 responding patients in the CMF arm. Median TTP was similar between the 2 treatments (4.1 months for capecitabine and 3.0 months for CMF). In addition, median OS was 19.6 months and 17.2 months for the capecitabine and CMF groups, respectively. Adverse-effect profiles were different, with a higher incidence of diarrhea (48% vs 22%) and hand-foot syndrome (43% vs 0%) in the capecitabine arm vs. more frequent alopecia (8% vs 19%) and grade 3 or 4 neutropenia (8% vs 41%) in patients receiving CMF [39]. 

The simplicity of administration of capecitabine led to further investigation of the drug in elderly patients with metastatic breast cancer [47]. Prior chemotherapy and/or hormonal treatment were permitted if it was not for metastatic disease. Capecitabine (1,500 mg/m^2^ per day in 2 divided doses on days 1 through 14) was given every 21 days until evidence of disease progression or unacceptable toxicity. Sixty-three patients were enrolled with a median age of 70 years (range 65-78 years) and Eastern Cooperative Oncology Group performance status of 0 to 2. Dose reduction by 25% was required in 17 patients for reasons, not specified in the abstract. The overall RR was 27% and stable disease was achieved in an additional 43%, after a median follow-up time of 16.3 months. The median TTP was 3.5 months. The most common toxicities were gastrointestinal and musculoskeletal related to hand-foot syndrome [47].

**Table 4:** Phase II trials of Capecitabine alone or in combination therapy in patients with advanced or metastatic breast cancer.

Author, year of publication	Number of patients	Chemotherapeutics used	Doses of drugs	% overall response (CR+PR)	mTTP Months	Median Survival Months
**First-line therapy for metastatic disease**						
Gradishar *et al.* [37], 2004	48	Capecitabine	- 1,650 mg/m^2^ per day (days 1-14)	51	10.6	29.9
		Paclitaxel	- 175 mg/m^2^ day 1			
Ghosn *et al.* [38], 2003	30	Capecitabine	- 1,650 mg/m^2^ per day (days 1-14)	68	9.3	NR
		Vinorelbine	- 25 mg/m ^2^ days 1 and 8			
O’Shaughnessy *et al.*	61	Capecitabine	- 2,510 mg/m^2^ per day (days 1-14)	30	4.1	19.6
[39], 2003	32	CMF	- CMF regimen	16	3.0	17.2
**Single-agent therapy in metastatic disease pre-treated with anthracyclines**						
Talbot *et al.* [40], 2002	22	Capecitabine	- 2,510 mg/m^2^ per day (days 1-14)	36	3.0	7.6
	19	Paclitaxel	-175 mg/m^2^ day 1	26	3.1	9.4
**Single-agent therapy in metastatic disease pre-treated with anthracyclines and taxanes**						
Blum *et al.* [41], 1999	162	Capecitabine	-2,510 mg/m^2^ per day (days 1-14)	20	3.1	12.8
Reichardt *et al.* [42], 2003	136	Capecitabine	-2,500 mg/m^2^ per day (days 1-14)	15	3.5	10.1
Fumoleau *et al.* [43], 2004	126	Capecitabine	-2,500 mg/m^2^ per day (days 1-14)	28	4.9	15.2
Blum *et al.* [44], 2001	75	Capecitabine	-2,510 mg/m^2^ per day (days 1-14)	29	3.2	12.2
Wist *et al.* [45], 2004	48	Capecitabine	-2,500 mg/m^2^ per day (days 1-14)	29	3.6	9.4
**Combination therapy in metastatic disease pre-treated with anthracyclines**						
Batista *et al.* [46], 2004	73	Capecitabine	- 2,000 mg/m^2^ per day (days 1-14)	52	8.1	16.5
		Paclitaxel	- 175 mg/m^2^ day 1			

*Abbreviations*: CR - complete response; PR - partial response; mTTP - median time to progression; NR - not reached. All capecitabine doses were divided equally and dosed twice daily. Regimens were administered every 3 weeks. CMF: cyclophosphamide 600 mg/m^2^, methotrexate 40 mg/m^2^, and fluorouracil 600 mg/m^2^ IV every 3 weeks.

The combination therapy with capecitabine as first line treatment for advanced or metastatic disease has been also evaluated. Capecitabine (1,650 mg/m^2^ per day divided in 2 doses) on days 1 through 14 and vinorelbine (25 mg/m^2^ IV) on days 1 and 8 were given every 21 days to 30 patients as first-line therapy for metastatic disease [38]. Neoadjuvant and/or adjuvant therapy had been received by 67% of the patients. An overall RR of 68% was achieved and median survival was not reached after 18 months of follow-up [25]. Capecitabine with paclitaxel also produced encouraging results as first-line therapy for metastatic breast cancer.

Capecitabine (1,650 mg/m^2^ per day divided in 2 doses) on days 1 through 14 with paclitaxel (175 mg/m ^2^ IV on day 1) was administered every 21 days. 77% of patients had received prior neoadjuvant and/or adjuvant chemotherapy. Of the 48 patients enrolled, objective responses were seen in 51%, with 17 patients experiencing a CR. Median OS was 29.9 months [38]. Although combination therapy has yielded positive results, larger studies are needed to further evaluate these combinations and others to determine their role in first-line therapy for metastatic disease.

The role of single agent capecitabine in the treatment of metastatic disease which is resistant to anthracyclines and/or taxanes has been investigated in several Phase II studies [40,41,42,43,44,45]. Resistance is defined as relapse within 6 months of completion of adjuvant therapy, initial response followed by progressive disease while receiving the same therapy, or progressive disease without response while receiving the same therapy. Treatment failure is defined as stable disease after a minimum of 4 cycles, CR or PR followed by progressive disease within 12 months of treatment, or disease recurrence 6 to 12 months after completion of adjuvant treatment [40,41,42,43,44].

The only single-agent comparative trial in previously treated patients was a small multicenter, randomized, Phase II study in patients with metastatic disease resistant to anthracyclines, either as adjuvant therapy or in the metastatic setting [40]. Patients were randomized to receive either capecitabine (2,510 mg/m^2^ per day in 2 divided doses) on days 1 through 14 every 21 days or paclitaxel (175 mg/m^2^ IV) on day 1 of every 21 days. Twenty two patients received capecitabine and 19 received paclitaxel. The primary end point was overall RR, and it was not significantly different between groups (36% for capecitabine and 26% for paclitaxel). CRs were seen in three capecitabine patients and none of those had received paclitaxel. Median TTP (3.0 months vs 3.1 months) and median OS (7.6 months vs 9.4 months) were similar between the capecitabine and paclitaxel groups. Tolerability profiles were different, with diarrhea (41% vs 16%), vomiting (41% vs 16%), and hand-foot syndrome (18% vs 0%) in the capecitabine group and alopecia (47% vs 0%) and musculoskeletal effects (26% vs 0%) in the paclitaxel group. Overall, grade 3 adverse effects were experienced in 23% of capecitabine patients and 58% of those who had received paclitaxel [40,45]. 

In patients with metastatic breast cancer previously treated with an anthracycline there is only a multicenter, multinational, open label, randomized, Phase III trial which compared a single agent docetaxel with a combination of docetaxel and capecitabine [36]. This combination is attractive because preclinical studies of taxanes support up regulation of thymidine phosphorylase in tumor tissue, and subsequent synergistic activity with capecitabine and paclitaxel or docetaxel [48]. Patients were enrolled if they had unresectable locally advanced and/or metastatic disease that was refractory to anthracyclines. Patients who had received docetaxel in either the adjuvant or metastatic setting were excluded, although prior paclitaxel treatment was allowed with no minimum interval between prior exposure and beginning of the current study.

The median TTP was the primary end point. Medical care utilization, safety, overall RR, OS, was secondary end points. Using the European Organisation for Research and Treatment of Cancer questionnaire, the quality of life was also evaluated. Randomization was made by country and stratification by whether or not paclitaxel had been a part of their previous treatment schedule. Treatment consisted of at least 6-weeks-oral capecitabine (2,500 mg/m^2^ per day in 2 divided doses within 30 minutes of a meal) on days 1 to 14 plus IV docetaxel (75 mg/m^2^) on day 1 of each 21-day cycle or single-agent docetaxel (100 mg/m^2^ IV) on day 1 of each 21-day cycle. Patients in the single-agent arm with mild hepatic impairment received a lower dose of docetaxel at 75 mg/m^2^. Additional dosage modification was made for toxicity while receiving therapy. Treatment was continued until disease progression or unacceptable toxicity occurred [36]. 

Between the two groups, the base line characteristics including sites of metastases, estrogen/ progesterone receptor status and age, were well balanced and a total of 511 patients were enrolled. The designed treatment was received by almost two thirds of both groups as second or third line therapy for metastatic disease. The median duration of treatment was 3.8 months for the combination group and 2.8 months for the docetaxel single-agent group. The group receiving the capecitabine/docetaxel combination had a median TTP of 6.1 months compared with 4.2 months for the group receiving docetaxel alone (P < 0.001). Median survival was more favourable in the combination group as well (14.5 months vs 11.5 months, P = 0.013). Objective tumor RR also favoured the combination arm, with 42% of patients achieving either a CR or PR, compared to 30% of patients in the docetaxel arm (P = 0.006). This was confirmed by an IRC, which reported a RR of 32% versus 23% for the combination and single-agent arms, respectively (P =0.025). The median time to treatment failure was 4 months for the capecitabine/docetaxel group and 2.8 months for the docetaxel group (P < 0.001). Gastrointestinal adverse effects (grade 3 or 4 stomatitis: 17.4% vs 5%; diarrhea: 14.4% vs 5.4%; nausea: 6% vs 2%) and hand-foot syndrome (grade 3: 24% vs 1%) were more common in the patients receiving combination therapy, while neutropenic fever (grade 3 or 4: 16% vs 21%) and arthralgia (grade 3 or 4 data not provided) was more common in the docetaxel group. Overall, grade 3 toxicities were more frequent in the combination group than in the single agent group (71% vs 49%). In the combination group, dosage reduction due to adverse effects was required in 4% for capecitabine alone, 10% for docetaxel alone, and 51% for both drugs. In the docetaxel arm, 36% of patients required a dosage reduction. A retrospective analysis examining the effects of that dose reduction, failed to show negative impact on efficacy. Additionally, no significant differences between the 2 treatment arms in terms of quality of life were reported by the patients on the questionnaires [36]. 

Taking this study into account, the FDA approved the combination of capecitabine and docetaxel for patients with progressive metastatic breast cancer after prior treatment with anthracycline based chemotherapy [9]. The dose regimen approved for this indication is the 1250 mg/m^2^ capecitabine administered orally twice a day for the first 2 weeks with 75 mg/m^2^ docetaxel as a 1-hour IV infusion on day 1 of each 3-week cycle, although the percentage of patients requiring dose adjustments may suggest that a lower starting dose yields better tolerability without compromising therapeutic benefit. This has not yet been evaluated in a clinical trial.

Following the results of this Phase III study, a non comparative Phase II study evaluating the combination of capecitabine and paclitaxel was conducted in 73 patients with advanced or metastatic breast cancer that had progressed following treatment with anthracyclines [46]. Patients were excluded if they had previously received paclitaxel, but were still enrolled if they had received docetaxel in either the adjuvant or metastatic setting. Oral capecitabine (2,000 mg/m^2^ per day in 2 divided doses) on days 1 to 14 plus IV paclitaxel (175 mg/m^2^) on day 1 of each 21-day cycle was administered. The overall RR was 52%, with 8 patients achieving a CR. The median TTP was 8.1 months with a median OS of 16.5 months. The most common nonhematologic toxicities of this combination regimen were hand-foot syndrome (42%), diarrhea (26%), and alopecia (30%) [46]. 

The uses of single agent capecitabine after disease progression following treatment with anthracyclines and taxanes for metastatic breast cancer has been evaluated in several non comparative Phase II trials (Table 3). Patients received 2,500 to 2,510 mg/m^2^ per day in 2 divided doses on days 1 through 14 of every 21-day cycle. Overall RR ranged from 15% to 29%, with stable disease in 31% to 46% of patients. Median OS ranged from 9.4 to 15.2 months. Tolerability has been favourable, with the majority of adverse effects being gastrointestinal (diarrhea, stomatitis, nausea, and vomiting) or related to hand-foot syndrome and fatigue. Hematologic toxicity and alopecia, commonly noted in anthracycline and taxane-based regimens, was infrequent [41,42,43,44,45]. As mentioned, single-agent capecitabine is also approved by the FDA for patients with metastatic breast cancer who have disease progression following treatment with paclitaxel and an anthracycline or in whom an anthracycline is contraindicated [9]. 

Data from the Brazil National Cancer Institute shows that approximately 30% of the new diagnosed patients with breast cancer have locally advanced disease. Since these patients are inoperable, a tumor reduction is often attempted using chemotherapy. Despite the fact that first line anthracycline based neoadjuvant chemotherapy is usually effective, about 30% of patients fail and there is not established second line treatment. Such a treatment using combination of radiation therapy and capecitabine was recently evaluated by Gaui *et al.* [49]. In this trial twenty-eight patients with inoperable locally advanced breast cancer refractory to first-line anthracycline based treatment were enrolled. Patients received radiation therapy (total dose 5,000 cGy) and concomitant capecitabine (850 mg/m^2^) twice daily for 14 days every 3 weeks. From 28 patients 23 were enabled operable (82%). The five remaining patients did not undergo surgery because of disease progression. The median clinical tumor size decreased from 80 cm^2^ to 49 cm^2^. Microscopic residual disease was observed in three patients (13%) and another patient achieved a complete pathologic response. The median number of involved lymph nodes was 2 and treatment was well tolerated with no grade 3 or 4 toxicity. The authors concluded that second-line neo-adjuvant treatment with radiation therapy and capecitabine is feasible, well tolerated, and effective in patients with locally advanced breast cancer refractory to primary anthracycline-based treatment. These results lead to the fact that a randomized study should compare radiotherapy alone with a combination between capecitabine and radiotherapy.

### Prostate cancer

Antitumor activity of capecitabine on prostate cancer was demonstrated in early xenograft studies in the PC-3 prostate cancer cell line in which was found a 77% inhibition of growth [50]. Moreover immunohistochemical analysis of prostate carcinoma tissue suggests high levels of thymidine phosphorylase in certain types of prostate [51]. The benefit from single-agent capecitabine was evaluated in a small group of patients with metastatic hormone resistant prostate cancer (HRPC) that had progressed after orchiectomy or medical castration [52]. This study was a Phase II non-comparative trial, included 25 patients and considered response based on prostate specific antigen (PSA) and clinical benefit as determined by a decrease in baseline pain. A PSA response was defined as a decrease of at least 50% from baseline and confirmed 4 weeks later by a second measurement in the absence of an increase in size of metastasis, appearance of new lesions, or clinical signs of disease progression. Oral capecitabine was scheduled at 2,500 mg/m^2^ per day in 2 divided doses on days 1 to 14 of every 21-day cycle. Patients had to have a PSA >3 times the upper limit of normal to be eligible. A PSA response was seen in 12% of patients, with a TTP of 18 to 35 weeks for these 3 patients. Ten patients required dose reduction secondary to hand-foot syndrome, diarrhea, hematologic toxicity, renal dysfunction, weight loss, vomiting, and spinal cord compression. Based on the poor RR in this trial, a Phase III trial of single-agent capecitabine was not undertaken [52]. Nevertheless, in vitro data support a possible inverse relationship between thymidine phosphorylase expression and PSA activity [51]. By enrolling only patients with higher PSA values in this trial, those who may have had the greatest benefit were excluded.

The combination effectiveness of docetaxel and capecitabine on metastatic HRPC was investigated on 23 patients in a phase II study [39]. Capecitabine (2,500 mg/m^2^ per day in 2 divided doses) was administered on days to 18 with IV docetaxel (135 mg/m^2^) on days 1, 8, and 15 of each 28-day cycle. A decrease in PSA of >50% was used to evaluate biological efficacy. The median PSA value of patients was 73.2 ng/mL and ranged between 0.93 and 2010.9 ng/mL. Biological response was observed in 41.2% of patients at the end of the second cycle and in 71.4% at the end of the fourth cycle. The most common adverse effects were anemia and gastrointestinal effects [53]. Additional trials are needed to determine the role of capecitabine alone and in combination for HRPC therapy.

### Renal cell cancer

In comparison with normal kidney tissue, thymidine phosphorylase was found in higher concentrations in kidney tumors, at a ratio of almost 7:1 [7]. In patients with renal cell carcinoma, the capecitabine was evaluated with the aim that this would interpret clinical benefits. In 26 patients with metastatic disease in whom immunotherapy had failed, a Phase II trial was performed to investigate the therapeutic benefit and toxicity of single agent capecitabine [54]. Capecitabine was dosed at 2,500 mg/m^2^ per day in 2 divided doses on days 1 to 14 of each 21-day cycle. Of the 23 patients evaluated for response, two experienced a PR, five had a minor response, and stable disease was seen in 13 patients. Median TTP was 6 months, with a median OS of 13 months. The rates of clinical benefit, defined as clinical response or stable disease, in patients who received capecitabine as second-line and third-line treatment, were 71.4% and 66.7%, respectively. Tolerability was favourable, with three patients experiencing grade 3 toxicity (hand-foot syndrome, two patients; anemia, onw patient) [54]. A second Phase II study of single agent capecitabine at the same dosage in a similar population was terminated after the first 14 patients had enrolled as it had failed to show either CR or PR. However, stable disease was seen in three of those patients for duration of 18 to 45 weeks [55]. 

Immunotherapy and chemotherapy together have been investigated as an effort to improve RR. In 24 patients with metastatic renal cell carcinoma capecitabine with interferon alfa 2a (INF-α2a) was evaluated as first line therapy [56]. Capecitabine (2,500 mg/m^2^ per day in 2 divided doses) was administered on days 1 to 14 of each 21-day cycle with SC (subcutaneous) IFN-α2a 6 million IU (MIU) 3 times per week. The partial RR was 25% and stable disease was achieved in an additional 33%. Median survival was 257 days, with a median TTP of 127 days [56]. The combination was also evaluated in another Phase II trail in 30 patients with metastatic renal cell carcinoma [57]. It was not stated in the study whether the treatment was first line. Therapy consisted of IFN-α2a 5 MIU/m^2^ SC daily on day 1 of weeks 1 and 4 and days 1, 3, and 5 of weeks 2 and 3 followed by 10 MIU/m^2^ SC daily on days 1, 3, and 5 of weeks 5 to 8; interleukin-2 10 MIU/m^2^ SC twice daily on days 3, 4, and 5 of weeks 1 and 4 followed by 5 MIU/m^2^ SC daily on days 1, 3, and 5 of weeks 2 and 3; capecitabine 2,000 mg/m^2^ per day in 2 divided doses on days 1 to 5 of weeks 5 to 8; and 13-*cis-*retinoic acid 35 mg/ m^2^ orally on days 1 to 7 of weeks 1 to 8. After a median follow-up of 8 months, all but three patients were alive. CRs were achieved in two patients, with a median response duration of >9 months. Eight patients had a PR, with a median response duration of >8 months. An additional 12 patients had a stable disease. The most common adverse effects were gastrointestinal, malaise, hand-foot syndrome, and dermatitis. No grade 4 toxicity was reported and only two patients had grade 3 toxicities (malaise, nausea, vomiting, and stomatitis) [57]. 

Phase II trials using capecitabine in combination with other chemotherapeutic agents are currently under way. The Cancer and Leukemia Group B combined capecitabine with gemcitabine to treat 55 patients with metastatic renal cell carcinoma [58]. Capecitabine (1,660 mg/m^2^ per day in 2 divided doses) was administered on days 1 through 21 with IV gemcitabine (1,000 mg/m^2^ per day) on days 1, 8, and 15 of each 28-day cycle. Prior systemic therapy had been administered in 42 patients, and 45 had undergone nephrectomy. The rate of PR was 15% for a median duration of 7.1 months. Median TTP was 5.1 months. Median survival had not been reached at the median follow-up of 5.5 months. The most common grade 3 or 4 toxicity was neutropenia in 40% of patients [58]. A docetaxel-capecitabine combination is currently being evaluated in a similar population [59]. The optimal combination regimen of capecitabine and immunotherapy and/or chemotherapy for renal cell carcinoma is still unknown.

### Ovarian cancer

Higher expression levels of thymidine phosphorylase are found in certain histologic types of ovarian cancer compared to healthy ovarian tissue. In a study, this ratio was 3:1 [7,60]. Moreover, xenograft models using the SK-OV-3 and Nakajima ovarian tumor cell lines demonstrated no tumor growth inhibition with the SK-OV-3 but 89% inhibition with the Nakajima ovarian tumor cell lines [60]. Due to the fact that the second and third line therapies for ovarian cancer are rather palliative, the encouraging preliminary data on its favourable toxicity profile made capecitabine a candidate for further investigation. The first Phase II trail of single agent capecitabine included patients with epithelial ovarian cancer who had received prior platinum based chemotherapy and relapsed within 12 months of their last chemotherapy [61]. A maximum of three prior regimens were allowed and all patients had measurable disease demonstrated radiologically and indicated by a serum CA125 >100 kU/L. Capecitabine (2,500 mg/m^2^ per day in 2 divided doses) for 14 days followed by 7 days of rest constituted the 21-day cycle. Response was determined by weekly CA125 measurements and was defined either as a 50% decrease from two previous, consistently elevated samples or >75% serial decrease over three samples. Twenty nine patients were enrolled and 29% met the CA125 criteria for response. The median progression-free survival was 3.7 months with an OS of 8.0 months. After 6 months of treatment, 28% remained without evidence of disease progression and 62% were still alive. Of the 14 patients with measurable disease, one had a complete radiologic response and five achieved stable disease. One patient experienced uncomplicated grade 4 leukopenia, while three patients had grade 3 vomiting and four had grade 3 hand-foot syndrome, resulting in two withdrawing from treatment [61]. 

A second Phase II trial enrolled patients who had received a median of four prior chemotherapy regimens for relapsed ovarian carcinoma (including both platinum and taxane regimens) [62]. Patients had to have unidimensionally measurable disease or CA125 >70 U/mL. The single-agent capecitabine dosing was identical to the previous study. CA125 response was defined as in the previous study, and measurable tumor response defined according to RECIST criteria, with the only modification being the additional presence of a normal CA125 value to qualify as CR [62,63]. Briefly, the RECIST criteria defines a response as a decrease of at least 30% in the sum of the longest diameter of targeted lesions compared with baseline measurements; a nonresponse is defined as <30% decrease or an increase in the sum of the longest diameter. Lesions measuring <10 mm by computed tomography scan or <20 mm by ultrasonography and also included cystic lesions, ascites, and patients in whom different imaging techniques were used for response assessment were defined as a non-measurable disease [63]. Of the 35 patients enrolled, 30 patients were evaluable for CA125 response and two responded, while 21 patients were evaluated using RECIST criteria and one had a PR. The duration of response for these three patients was 9, 19, and 21 weeks. Using the intent-to-treat population, the estimated median progression free survival and median OS were 2.3 and 7.1 months, respectively. No grade 4 toxicity was experienced; however, grade 3 hand-foot syndrome and diarrhea was seen in 17% and in 9%, respectively [62]. 

There is a scarcity of studies that evaluate combination therapy with capecitabine in ovarian cancer. A small Phase I/II trial of epirubicin-carboplatin-capecitabine in 11 ovarian cancer patients who had relapsed after platinum therapy evaluated the MTD of the combination; tumor response was a secondary end point [64].The treatment consisted of epirubicin (50 mg/m^2^ IV bolus) and carboplatin (AUC 5 IV on day 1) with oral capecitabine (750 mg/m^2^ per day, level 1, or 1,000 mg/m^2^ per day, level 2) in 2 divided doses given daily for a 21-day cycle or 1,000 mg/m^2^ per day for days 1 to 14 of a 21-day cycle (level 3). Across all dosing levels, six patients achieved a PR or CR, and an additional patient had a stable disease [64]. Larger studies including that combination and others, are needed to define clinical benefits.

### Pancreatic cancer

In pancreatic cancer, 5-FU has been extensively investigated showing small impact on OS and disease control [65]. Capecitabine, both as a single agent and in combination with other chemotherapeutic agents, has also been evaluated in this tumor type [66,67,68,69,70,71,72]. Response in terms of clinical benefit is used to evaluate patients with pancreatic cancer due to the palliative nature of therapy and the disease-related symptoms including pain, functional impairment, and anorexia that are often present at later stages. Clinical benefit response evaluates pain control in terms of intensity and analgesic consumption, functional impairment using the Karnofsky performance status (KPS), and changes in body weight. Responses are defined as positive if a predetermined percentage improvement from baseline is observed (usually 50%) and maintained for at least 4 weeks. Worsening from baseline values defines a negative response [66]. 

In 42 patients with advanced or metastatic pancreatic cancer, capecitabine was evaluated as a single agent [66]. Exclusion criteria included prior chemotherapy in the adjuvant or metastatic disease settings. Capecitabine was scheduled at 2,500 mg/m^2^ per day in 2 divided doses on the first 2 weeks of each 3-week cycle. Tumors were assessed at 6-week intervals and pain assessment diaries were reviewed at each visit. The primary end point was overall RR based on tumor measurement or clinical benefit response. A responder was defined as a patient with measurable disease who had an objective response, a patient with no measurable disease who experienced complete resolution and disappearance of all visible disease, or a patient with assessable disease who had evidence of stable or improved residual disease (PR) with a positive clinical benefit response. All but one of the 42 patients enrolled had measurable disease. PR was seen in three of these patients, with duration of response of 208, 260, and 566 days. An additional patient met response criteria based on improvement in residual disease and positive clinical benefit response. Stable disease was achieved in 41% for a median of 127 days. Median survival time based on Kaplan-Meier estimate was 182 days. With respect to the clinical benefit, positive responses were seen in pain intensity for 29% and analgesic usage in 12%. Stable disease was seen in 60% for pain intensity, 55% for analgesic usage, and 90% for performance status. Overall, the clinical benefit response rate was 24%. Adverse effects were predominantly gastrointestinal events (nausea, diarrhea, and vomiting) and hand-foot syndrome, with two patients experiencing grade 4 diarrhea [66]. 

In patients with pancreatic cancer the combination therapy with capecitabine and gemcitabine has also been studied. The combination was estimated in a Phase I/II dose finding trial and had as a result 1 CR and 4 PRs among 27 patients enrolled [67]. In patients with metastatic pancreatic adenocarcinoma the benefit of that combination was compared with single-dose gemsitabine [68]. The regimen in this trial was more intense than in the previous trial considering gemcitabine (2,200 mg/m ^2^ IV on day 1 as a 30-minute infusion) with or without capecitabine (2,500 mg/m^2^ per day in 2 divided doses) on days 1 through 7 of each 2 week cycle. Exclusion criteria included locally advanced disease, total bilirubin <1.5 mg/dl, or transaminases levels < 2 times the upper limit of normal. Previous adjuvant therapy with fluoropyrimidine and/or radiation was permitted. Further stratification was made based on KPS (90%-100% vs 50-80%) and prior adjuvant therapy (chemotherapy and/or radiation vs no prior treatment). A total of 83 patients were enrolled, with prognostic factors including histologic grade of tumor, site of metastases, KPS, and prior surgery equally distributed between the treatment groups. The progression-free survival and the median OS were similar in the combination and gemcitabine groups (5.1 vs. 4.0 months and 9.5 vs. 8.2 months, respectively). IRC assessment of clinical RR was 17.1% versus 14.3% in the combination and single-agent groups, with all responses being partial, occurring within 3 months of treatment with median duration of 5.5 to 5.8 months. Stable disease was achieved in 56% of the combination patients and 43% of the gemcitabine patients. The clinical benefit response was evaluated in 30 patients in the single agent arm and 31 patients receiving combination therapy. Improvement in pain and/or performance status was reported by 48.3% of the patients in the capecitabine/gemcitabine group and 33% of the patients in the gemcitabine group. Gastrointestinal symptoms (stomatitis: 5.1% vs 25 %; diarrhea: 20.5% vs 35%) and hand-foot syndrome (26% vs 0%) were more common in the capecitabine arm. Myelo-suppression was the most common toxicity in both arms. Only a patient in each arm stopped treatment due to adverse effects [68]. 

The same combination of chemotherapeutic agents using fewer drugs per week was evaluated in patients with inoperable or metastatic adenocarcinoma who were chemotherapy and radiotherapy naïve [69]. Capecitabine (1,300 mg/m^2^) was given daily in 2 divided doses on days 1 through 14 of each 3-week cycle with gemcitabine (1,000 mg/m^2^ IV) on days 1 and 8 as a 30-minute infusion. Fifty-three patients were enrolled,

85% of who had stage IV disease. The primary end point was efficacy, measured by RR. PRs were seen in 18.9% of patients, all of whom had stage IV disease, while 41.5% had stable disease. The median duration of response was 3 months with TTP of 6.5 months. Overall median survival was 10 months in patients with stage III disease and 8.0 months in patients with stage IV disease. There was no improvement in terms of performance status with the majority of patients having no change and 14 reporting a decrease in capability of doing their daily activities. Among 43 patients that reported pain, improvement was seen in 53% of them, while 7% reported deterioration. Hematologic toxicity was the most common grade 3 or 4 adverse effect [69]. 

The combination of gemsitabine with dose escalated 14 days Capecitabine in patients with locally advanced pancreatic cancer has also been investigated [69]. Prior systemic chemotherapy, other than that given concurrently with radiation therapy, was not accepted and the patients had at least one measurable disease, and adequate organ functions. The 45 patients enrolled were treated with gemsitabine 1,000 mg/m^2^ IV on days 1, 8 and capecitabine 1,000 mg/m^2^ twice a day PO on days 1-14, in 21-day cycles. The objective RR among 45 patients was 40.0% (95% CI; 25.1-54.9), including 1 CR (2.2%). The median TTP and OS were 5.4 months (95% CI; 1.8-9.0) and 10.4 months (95% CI; 6.2-14.5), respectively. The most frequent, grade 3-4, non-hematologic toxicity was hand-foot syndrome (6.7%). The authors have concluded that the combination is well tolerated and offers an encouraging activity in the treatment of advanced pancreatic cancer. 

The addition of another agent (cisplatin or docetaxel) to capecitabine and gemcitabine combination has also been investigated [70,71]. These combinations were investigated in small Phase II studies that enrolled 35 to 40 patients with advanced or metastatic pancreatic cancer. Responses were mostly PRs and were achieved in 30% to 40% with median survival of 7.5 to 10.5 months [70,71]. In addition, a randomized, multicenter trial comparing capecitabine combinations with either oxaliplatin or gemcitabine with oxaliplatin/gemcitabine in a similar patient population is being conducted as an effort to better define the role of capecitabine in the treatment of pancreatic cancer [72]. 

The effectiveness of combined radiation therapy with capecitabine in patients with locally advanced pancreatic cancer has also been evaluated [73]. Twenty patients with locally advanced pancreatic cancer have received 50.4 Gy radiation therapy, daily fraction 1.8 Gy, 25 fractions in total, with capecitabine 1,600 mg/m^2^ on Monday through Friday for 6 weeks). After capecitabine-radiation therapy, stable and responding patients received capecitabine (2,000 mg/m^2^) for 14 days every 3 weeks till progression. Restaging was performed every 9 weeks. Among patients, 4 (20%) had a partial response and 13 (65%) had stable disease. Two patients underwent surgical resection (10%). The 6-month survival rate was 84%, and the 1-year survival was 58%. Grade>or=3 toxicities included nausea/vomiting (5%), thrombosis (5%), hyperbilirubinemia (5%), and grade 3 gastrointestinal bleeding (5%). The authors have concluded that capecitabine-radiation therapy is an effective, tolerable, and convenient alternative to an infusional 5-fluorouracil regimen for patients with locally advanced pancreatic cancer. 

## Conclusions

In the Unites States the capecitabine is currently the only oral 5-FU pro-drug approved for use. In patients with metastatic colorectal cancer, the capecitabine is as effective as 5-FU having a toxicity profile that consists most commonly of gastrointestinal and dermatologic side-effects. In patients with metastatic colorectal and previously treated breast cancer the effectiveness of drug has been tested in large trials. The clinical evidence of those trials led the FDA to approve its use in both populations. In prostate, renal cell, ovarian and pancreatic cancers, the majority of evidence is from smaller, Phase II trials, and efficacy has been mostly in the form of PRs and stable disease. Combination therapy in these patients appears to be more beneficial than single-agent capecitabine; however, the specific combination and dosage regimens of chemotherapeutic and immunotherapeutic agents are yet to be ascertained. Additionally, the frequent need for capecitabine dosage adjustment due to adverse effects in both colorectal and breast cancer populations suggests that a lower starting dose may be beneficial.
